# Supplementary material for: Oxytocin receptors influence the development and maintenance of social behavior in zebrafish (Danio rerio)
Source: Sci Rep. 2022 Mar 12;12:4322. doi: 10.1038/s41598-022-07990-y (PMC8918347; doi:10.1038/s41598-022-07990-y)
Supplement: Supplementary file 1 — Supplementary Information. [file 41598_2022_7990_MOESM1_ESM.pdf]

Oxytocin receptors influence the development and maintenance of social behavior in zebrafish (*Danio rerio*).

Anja Gemmer[1], Kristina Mirkes[1], Lukas Anneser[1], Tim Eilers[1], Caroline Kibat[2,3], Ajay Mathuru[2,3,4], Soojin Ryu[5], Erin Schuman[1*]

1: Max Planck Institute for Brain Research, Germany

2: Department of Physiology, YLL School of Medicine, National University of Singapore, Singapore

3: Yale-NUS College, 12 College Avenue West, Singapore

4: Institute of Molecular and Cell Biology, A*STAR, Singapore

5: Living Systems Institute & College of Medicine and Health, University of Exeter, UK

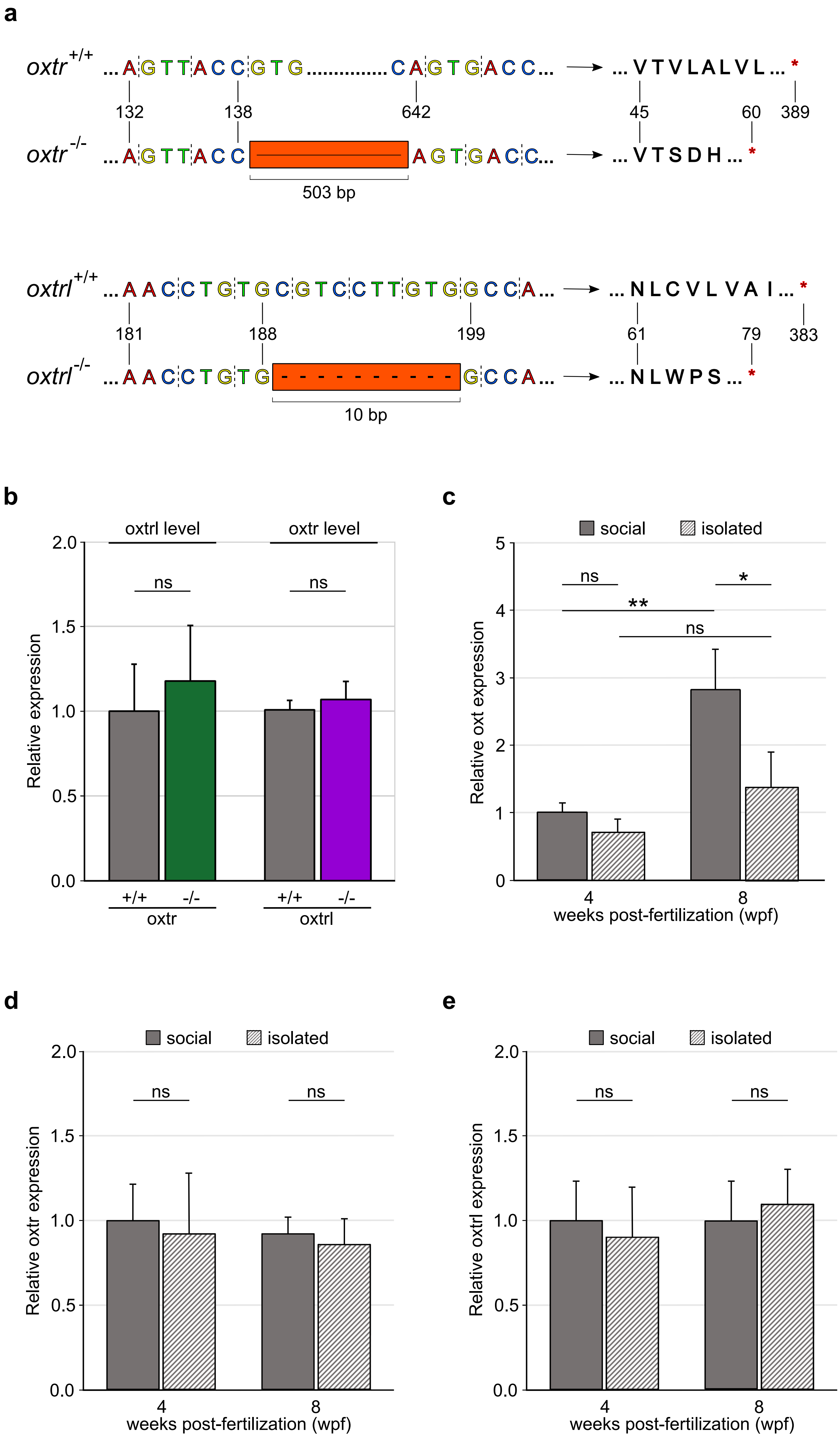

a
oxtr+/+ ... A G T T A C C G T G .... C A G T G A C C ... → ... V T V L A L V L ... *
132 138 642 45 60 389
oxtr-/- ... A G T T A C C A G T G A C C ... → ... V T S D H ... *
503 bp
oxtrl+/+ ... A A C C T G T G C G T C C T T G T G G C C A ... → ... N L C V L V A I ... *
181 188 199 61 79 383
oxtrl-/- ... A A C C T G T G - - - - - - - - - - G C C A ... → ... N L W P S ... *
10 bp
b
oxtrl level
oxtr level
ns
ns
Relative expression
2.0
1.5
1.0
0.5
0.0
+/+ -/-
oxtr
+/+ -/-
oxtrl
c
social
isolated
ns
*
**
ns
Relative oxt expression
5
4
3
2
1
0
4
8
weeks post-fertilization (wpf)
d
social
isolated
ns
ns
Relative oxtr expression
2.0
1.5
1.0
0.5
0.0
4
8
weeks post-fertilization (wpf)
e
social
isolated
ns
ns
Relative oxtrl expression
2.0
1.5
1.0
0.5
0.0
4
8
weeks post-fertilization (wpf)

Supplementary Figure S1: Details of the CRISPR/Cas9 generated KO, potential compensation and effect of isolation on *oxt, oxtr* and *oxtrl* transcription.

(a) A 503 base pair deletion in the *oxtr* gene and a 10 base pair deletion in the *oxtrl* gene caused frameshifts leading to premature stop codons. The resulting mutant Oxtr protein size is 59 amino acids (wild-type Oxtr protein is 388 amino acids) and mutant Oxtrl protein size is 78 amino acids (wild-type Oxtrl protein is 382 amino acids). From the different receptor parts (N- and C-terminus, seven transmembrane helices, three intracellular and three extracellular loops) only the N-terminus and parts of the first transmembrane helix were present in mutant Oxtr and Oxtrl, respectively.

(b) The knocked-out receptor was not compensated by enhanced expression of the intact cognate receptor (Oxtrl in *oxtr*$^{-/-}$, Oxtr in *oxtrl*$^{-/-}$). Asterisks represent the statistical comparison of relative gene expression between wild-type and mutant genotype. $n_{(oxtr^{+/+},\ 8\ wpf)} = 5$, $n_{(oxtr^{-/-},\ 8\ wpf)} = 6$, $n_{(oxtrl^{+/+},\ 8\ wpf)} = 6$, $n_{(oxtrl^{-/-},\ 8\ wpf)} = 4$. Kruskal-Wallis-Test: $p_{(8wpf)} = 8.80 \times 10^{-1}$, Wilcoxon rank-sum test for wild-type vs. mutant genotype: $p_{(oxtr^{+/+} \Leftrightarrow oxtr^{-/-})} = 9.31 \times 10^{-1}$, $p_{(oxtrl^{+/+} \Leftrightarrow oxtrl^{-/-})} = 9.31 \times 10^{-1}$.

(c) Isolation rearing led to significantly decreased *oxt* expression at 8 wpf. Under social conditions, wild-type zebrafish expressed Oxytocin at significantly higher levels with increasing age. This effect is significantly reduced after isolation rearing. Asterisks represent the statistical comparison of rearing condition or age. $n_{(social\ wildtype,\ 4wpf)} = 5$, $n_{(isolated\ wildtype,\ 4wpf)} = 6$, $n_{(social\ wildtype,\ 8wpf)} = 11$, $n_{(isolated\ wildtype,\ 8wpf)} = 10$. Kruskal-Wallis-Test: $p_{(wildtype)} = 8.00 \times 10^{-4}$, Wilcoxon rank-sum test for social vs. isolation rearing: $p_{(wildtype,\ 4wpf)} = 3.29 \times 10^{-1}$, $p_{(wildtype,\ 8wpf)} = 1.34 \times 10^{-2}$. Wilcoxon rank-sum test for 4wpf vs. 8wpf: $p_{(social\ wildtype)} = 1.83 \times 10^{-3}$, $p_{(isolated\ wildtype)} = 3.29 \times 10^{-1}$.

(d) The expression of *oxtr* was not altered by isolation rearing in both 4- and 8-week old wildtype zebrafish. Asterisks represent the statistical comparison of relative gene expression between wild-type and mutant genotype. $n_{(social\ wildtype,\ 4wpf)} = 5$, $n_{(isolated\ wildtype,\ 4wpf)} = 4$, $n_{(social\ wildtype,\ 8wpf)} = 7$, $n_{(isolated\ wildtype,\ 8wpf)} = 8$. Kruskal-Wallis-Test: $p_{(wildtype)} = 9.90 \times 10^{-1}$, Wilcoxon rank-sum test for social vs. isolation rearing: $p_{(wildtype,\ 4wpf)} = 9.33 \times 10^{-1}$, $p_{(wildtype,\ 8wpf)} = 9.33 \times 10^{-1}$.

(e) Similarly, the expression of *oxtrl* remained unaffected by isolation rearing at 4 and 8 wpf. Asterisks represent the statistical comparison of relative gene expression between wild-type and mutant genotype. $n_{(social\ wildtype,\ 4wpf)} = 5$, $n_{(isolated\ wildtype,\ 4wpf)} = 4$, $n_{(social\ wildtype,\ 8wpf)} = 7$, $n_{(isolated\ wildtype,\ 8wpf)} = 8$. Kruskal-Wallis-Test: $p_{(wildtype)} = 9.91 \times 10^{-1}$, Wilcoxon rank-sum test for social vs. isolation rearing: $p_{(wildtype,\ 4wpf)} = 9.05 \times 10^{-1}$, $p_{(wildtype,\ 8wpf)} = 9.05 \times 10^{-1}$.

Number of replicates (n) and excluded n can be found in Supplementary Table S1, significance values in Supplementary Table S2. Significance is reported as *$p<0.05$, **$p<0.01$, ***$p<0.001$.

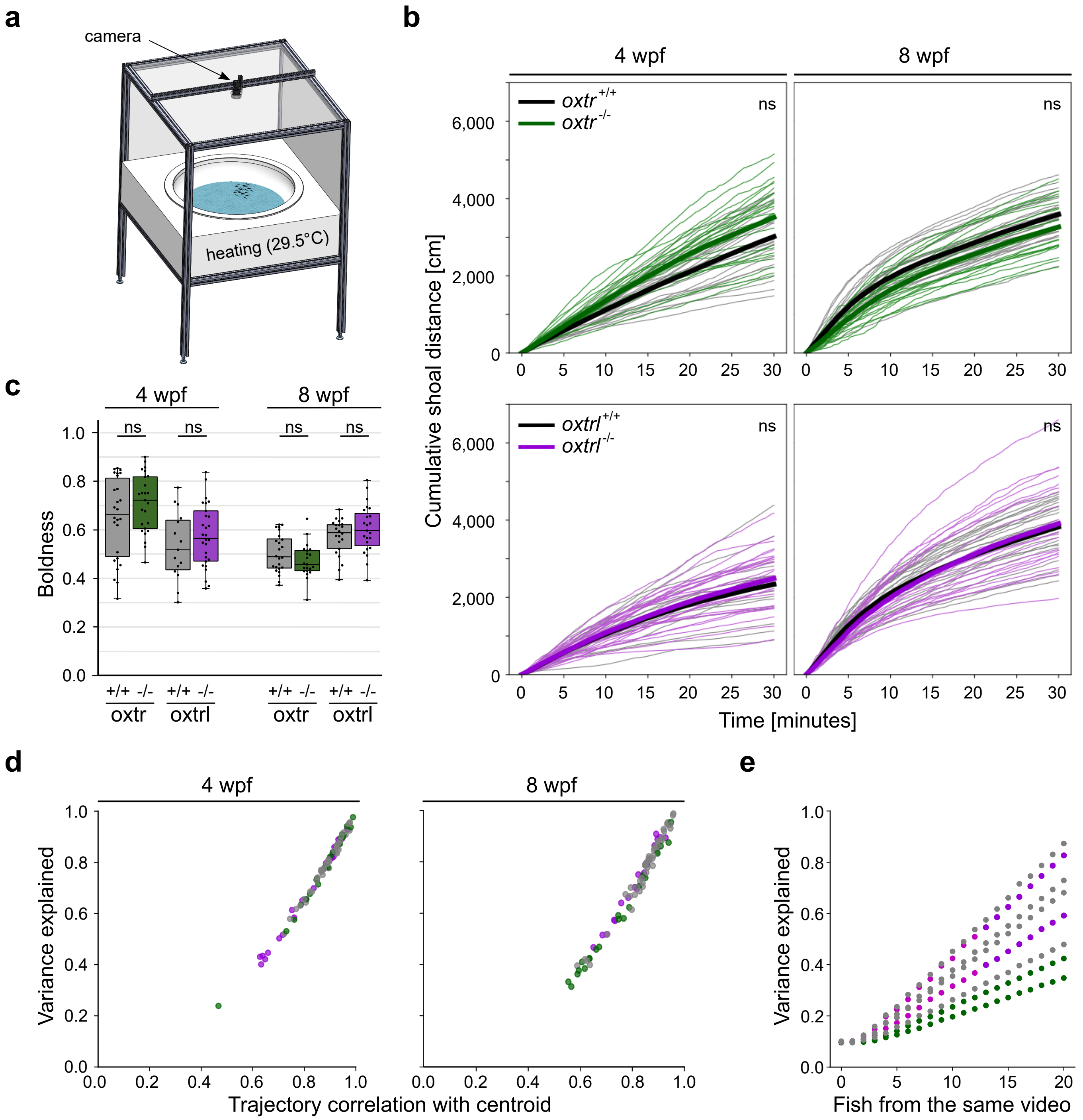

a
camera
heating (29.5°C)
b
4 wpf
8 wpf
oxtr+/+
oxtr-/-
oxtrl+/+
oxtrl-/-
ns
Cumulative shoal distance [cm]
Time [minutes]
c
Boldness
+/+ -/-
oxtr
oxtrl
d
Variance explained
Trajectory correlation with centroid
e
Fish from the same video

Supplementary Figure S2: Shoaling setup details, cumulative shoal distance, boldness and variance explained as polarization parameter.

(a) The behavioral chamber for the shoaling experiments was composed of a round tank, surrounded by 29.5°C warm water. White LEDs in the ceiling provided illumination for the camera positioned above.

(b) Knocking-out one of the Oxytocin receptors did not lead to significant changes in the cumulative shoal distance at both 4 and 8 wpf. Number of shoals (containing 20 fish each) per group: $n_{(oxtr^{+/+},\ 4wpf)} = 26$, $n_{(oxtr^{-/-},\ 4wpf)} = 25$, $n_{(oxtrl^{+/+},\ 4wpf)} = 15$, $n_{(oxtrl^{-/-},\ 4wpf)} = 28$, $n_{(oxtr^{+/+},\ 8wpf)} = 24$, $n_{(oxtr^{-/-},\ 8wpf)} = 21$, $n_{(oxtrl^{+/+},\ 8wpf)} = 22$, $n_{(oxtrl^{-/-},\ 8wpf)} = 23$. Kruskal-Wallis-Test for cumulative shoal distance: $p_{(4wpf)} = 2.00 \times 10^{-4}$, $p_{(8wpf)} = 5.25 \times 10^{-2}$, Wilcoxon rank-sum test for the cumulative shoal distance of wild-type vs. mutant genotype: $p_{(oxtr^{+/+} \Leftrightarrow oxtr^{-/-},\ 4wpf)} = 8.01 \times 10^{-2}$, $p_{(oxtrl^{+/+} \Leftrightarrow oxtrl^{-/-},\ 4wpf)} = 4.52 \times 10^{-1}$, $p_{(oxtr^{+/+} \Leftrightarrow oxtr^{-/-},\ 8wpf)} = 1.82 \times 10^{-1}$ $p_{(oxtrl^{+/+} \Leftrightarrow oxtrl^{-/-},\ 8wpf)} = 9.37 \times 10^{-1}$.

(c) Boldness, the willingness of a fish to enter open water, was not altered in either *oxtr* $^{-/-}$ or *oxtrl* $^{-/-}$. Each dot represents one shoal. Asterisks represent the statistical comparison between mutant and wild-type genotype at different ages. Number of shoals (containing 20 fish each) per group: $n_{(oxtr^{+/+},\ 4wpf)} = 26$, $n_{(oxtr^{-/-},\ 4wpf)} = 25$, $n_{(oxtrl^{+/+},\ 4wpf)} = 15$, $n_{(oxtrl^{-/-},\ 4wpf)} = 28$, $n_{(oxtr^{+/+},\ 8wpf)} = 24$, $n_{(oxtr^{-/-},\ 8wpf)} = 21$, $n_{(oxtrl^{+/+},\ 8wpf)} = 22$, $n_{(oxtrl^{-/-},\ 8wpf)} = 23$. Kruskal-Wallis-Test for boldness: $p_{(4wpf)} = 1.20 \times 10^{-3}$, $p_{(8wpf)} = 8.54 \times 10^{-6}$, Wilcoxon rank-sum test for the boldness of wild-type vs. mutant genotype: $p_{(oxtr^{+/+} \Leftrightarrow oxtr^{-/-},\ 4wpf)} = 3.66 \times 10^{-1}$, $p_{(oxtrl^{+/+} \Leftrightarrow oxtrl^{-/-},\ 4wpf)} = 3.66 \times 10^{-1}$, $p_{(oxtr^{+/+} \Leftrightarrow oxtr^{-/-},\ 8wpf)} = 2.71 \times 10^{-1}$ $p_{(oxtrl^{+/+} \Leftrightarrow oxtrl^{-/-},\ 8wpf)} = 2.71 \times 10^{-1}$.

(d) The variance explained correlates with the trajectory correlation with centroid, making the variance explained a useful polarization parameter.

(e) Adding more fish of the same video, while maintaining a group size of 20 with trajectories chosen from random videos, increased the variance explained – indicating its suitability as polarization parameter. Shown are two exemplar replicates per genotype group, color-coded in grey (*oxtr* $^{+/+}$ and *oxtrl* $^{+/+}$), green (*oxtr* $^{-/-}$) and purple (*oxtrl* $^{-/-}$).

Number of replicates (n) and excluded n can be found in Supplementary Table S1, significance values in Supplementary Table S2. Significance is reported as *p<0.05, **p<0.01, ***p<0.001.

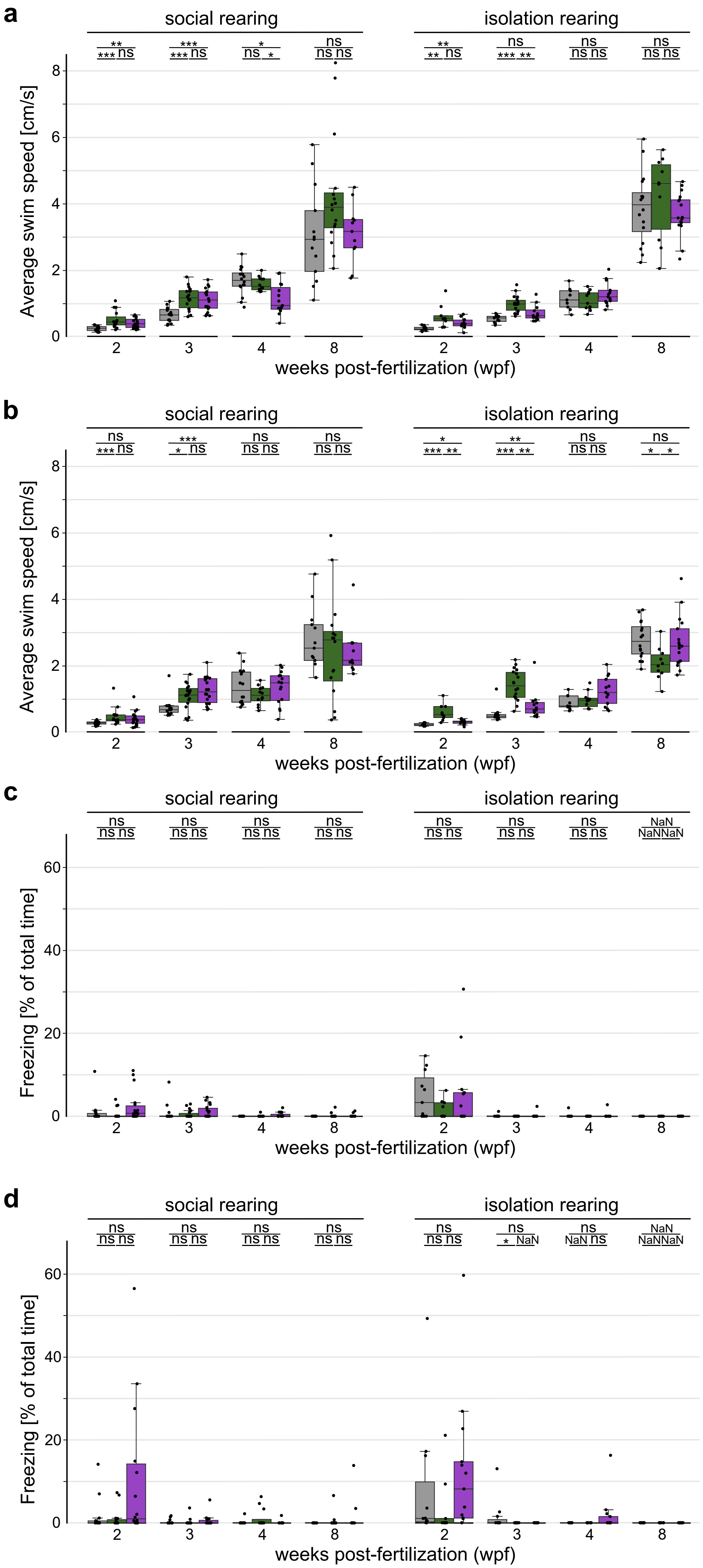

a
b
c
d
social rearing
isolation rearing
Average swim speed [cm/s]
Freezing [% of total time]
weeks post-fertilization (wpf)
2
3
4
8

Supplementary Figure S3: Swim kinematics in absence and presence of stimulus fish in the social preference test

(a) In the absence of stimulus fish (habituation period) the average swim speed of *oxtr* $^{-/-}$ fish was significantly increased at 2 and 3 wpf after both social and isolation rearing. This effect was also present in *oxtrl* $^{-/-}$ fish of the same age, with exception of 3-week-old *oxtrl* $^{-/-}$ after isolation rearing. In contrast, socially reared *oxtrl* $^{-/-}$ showed significantly reduced swimming speed at 4 wpf. Knocking out one of the Oxytocin receptors did not alter the swimming speed of isolation reared 4-week-old fish and 8-week old fish independent of the rearing condition. n of experimental fish: see below. Kruskal-Wallis-Test: $p_{(social\ 2wpf)} = 3.49 \times 10^{-4}$, $p_{(social\ 3wpf)} = 1.97 \times 10^{-4}$, $p_{(social\ 4wpf)} = 4.71 \times 10^{-3}$, $p_{(social\ 8wpf)} = 9.84 \times 10^{-2}$, $p_{(isolated\ 2wpf)} = 3.48 \times 10^{-4}$, $p_{(isolated\ 3wpf)} = 3.22 \times 10^{-5}$, $p_{(isolated\ 4wpf)} = 3.86 \times 10^{-1}$, $p_{(isolated\ 8wpf)} = 2.70 \times 10^{-1}$, Post-hoc Wilcoxon rank-sum test for social 2 wpf: $p_{(social\ 2wpf,\ wt \Leftrightarrow oxtr^{-/-})} = 6.69 \times 10^{-4}$, $p_{(social\ 2wpf,\ wt \Leftrightarrow oxtrl^{-/-})} = 3.81 \times 10^{-3}$, $p_{(social\ 2wpf,\ oxtr^{-/-} \Leftrightarrow oxtrl^{-/-})} = 2.86 \times 10^{-1}$, Post-hoc Wilcoxon rank-sum test for social 3 wpf: $p_{(social\ 3wpf,\ wt \Leftrightarrow oxtr^{-/-})} = 6.33 \times 10^{-4}$, $p_{(social\ 3wpf,\ wt \Leftrightarrow oxtrl^{-/-})} = 8.33 \times 10^{-4}$, $p_{(social\ 3wpf,\ oxtr^{-/-} \Leftrightarrow oxtrl^{-/-})} = 6.92 \times 10^{-1}$, Post-hoc Wilcoxon rank-sum test for social 4 wpf: $p_{(social\ 4wpf,\ wt \Leftrightarrow oxtr^{-/-})} = 2.36 \times 10^{-1}$, $p_{(social\ 4wpf,\ wt \Leftrightarrow oxtrl^{-/-})} = 1.25 \times 10^{-2}$, $p_{(social\ 4wpf,\ oxtr^{-/-} \Leftrightarrow oxtrl^{-/-})} = 2.06 \times 10^{-2}$, Post-hoc Wilcoxon rank-sum test for isolated 2 wpf: $p_{(isolated\ 2wpf,\ wt \Leftrightarrow oxtr^{-/-})} = 2.49 \times 10^{-3}$, $p_{(isolated\ 2wpf,\ wt \Leftrightarrow oxtrl^{-/-})} = 3.21 \times 10^{-3}$, $p_{(isolated\ 2wpf,\ oxtr^{-/-} \Leftrightarrow oxtrl^{-/-})} = 8.25 \times 10^{-2}$, Post-hoc Wilcoxon rank-sum test for isolated 3 wpf: $p_{(isolated\ 3wpf,\ wt \Leftrightarrow oxtr^{-/-})} = 6.48 \times 10^{-5}$, $p_{(isolated\ 3wpf,\ wt \Leftrightarrow oxtrl^{-/-})} = 6.94 \times 10^{-2}$, $p_{(isolated\ 3wpf,\ oxtr^{-/-} \Leftrightarrow oxtrl^{-/-})} = 8.34 \times 10^{-3}$,

(b) Knocking out one of the Oxytocin receptors leads to increased swim speed in presence of stimulus fish at 2 and 3 wpf after both social and isolation rearing. Except for a decrease in 8-week-old *oxtr* $^{-/-}$ after isolation rearing, the average swim speed was not altered in both *oxtr* $^{-/-}$ and *oxtrl* $^{-/-}$ at 4 and 8 wpf. n of test fish: see below. Kruskal-Wallis-Test: $p_{(social\ 2wpf)} = 2.08 \times 10^{-3}$, $p_{(social\ 3wpf)} = 7.97 \times 10^{-4}$, $p_{(social\ 4wpf)} = 3.04 \times 10^{-1}$, $p_{(social\ 8wpf)} = 6.61 \times 10^{-1}$, $p_{(isolated\ 2wpf)} = 6.03 \times 10^{-5}$, $p_{(isolated\ 3wpf)} = 8.11 \times 10^{-6}$, $p_{(isolated\ 4wpf)} = 1.19 \times 10^{-1}$, $p_{(isolated\ 8wpf)} = 1.23 \times 10^{-2}$, Post-hoc Wilcoxon rank-sum test for social 2 wpf: $p_{(social\ 2wpf,\ wt \Leftrightarrow oxtr^{-/-})} = 5.67 \times 10^{-4}$, $p_{(social\ 2wpf,\ wt \Leftrightarrow oxtrl^{-/-})} = 6.17 \times 10^{-2}$, $p_{(social\ 2wpf,\ oxtr^{-/-} \Leftrightarrow oxtrl^{-/-})} = 3.38 \times 10^{-1}$, Post-hoc Wilcoxon rank-sum test for social 3 wpf: $p_{(social\ 3wpf,\ wt \Leftrightarrow oxtr^{-/-})} = 1.46 \times 10^{-2}$, $p_{(social\ 3wpf,\ wt \Leftrightarrow oxtrl^{-/-})} = 6.33 \times 10^{-4}$, $p_{(social\ 3wpf,\ oxtr^{-/-} \Leftrightarrow oxtrl^{-/-})} = 3.04 \times 10^{-1}$, Post-hoc Wilcoxon rank-sum test for isolated 2 wpf: $p_{(isolated\ 2wpf,\ wt \Leftrightarrow oxtr^{-/-})} = 7.98 \times 10^{-4}$, $p_{(isolated\ 2wpf,\ wt \Leftrightarrow oxtrl^{-/-})} = 1.27 \times 10^{-2}$, $p_{(isolated\ 2wpf,\ oxtr^{-/-} \Leftrightarrow oxtrl^{-/-})} = 1.95 \times 10^{-3}$, Post-hoc Wilcoxon rank-sum test for isolated 3 wpf: $p_{(isolated\ 3wpf,\ wt \Leftrightarrow oxtr^{-/-})} = 1.43 \times 10^{-4}$, $p_{(isolated\ 3wpf,\ wt \Leftrightarrow oxtrl^{-/-})} = 7.40 \times 10^{-3}$, $p_{(isolated\ 3wpf,\ oxtr^{-/-} \Leftrightarrow oxtrl^{-/-})} = 1.34 \times 10^{-3}$, Post-hoc Wilcoxon rank-sum test for isolated 8 wpf: $p_{(isolated\ 8wpf,\ wt \Leftrightarrow oxtr^{-/-})} = 1.23 \times 10^{-2}$, $p_{(isolated\ 8wpf,\ wt \Leftrightarrow oxtrl^{-/-})} = 5.77 \times 10^{-1}$, $p_{(isolated\ 8wpf,\ oxtr^{-/-} \Leftrightarrow oxtrl^{-/-})} = 3.35 \times 10^{-2}$.

(c) Freezing behavior in the absence of conspecifics was not significantly influenced by the knock-out of one of the Oxytocin receptors at 2 to 8 wpf after both social and isolation rearing. n of test fish: see below. Kruskal-Wallis-Test: $p_{(social\ 2wpf)} = 6.57 \times 10^{-2}$, $p_{(social\ 3wpf)} = 2.71 \times 10^{-1}$, $p_{(social\ 4wpf)} = 6.25 \times 10^{-2}$, $p_{(social\ 8wpf)} = 2.91 \times 10^{-1}$, $p_{(isolated\ 2wpf)} = 4.05 \times 10^{-1}$, $p_{(isolated\ 3wpf)} = 4.48 \times 10^{-1}$, $p_{(isolated\ 4wpf)} = 5.67 \times 10^{-1}$, $p_{(isolated\ 8wpf)}$ = NaN (all values equal).

(d) In the presence of stimulus fish, freezing behavior was not altered in 2 to 8-week old *oxtr*$^{-/-}$ and *oxtrl*$^{-/-}$ after both social and isolation rearing. n of test fish: see below. Kruskal-Wallis-Test: $p_{(social\ 2wpf)} = 5.36 \times 10^{-2}$, $p_{(social\ 3wpf)} = 4.79 \times 10^{-1}$, $p_{(social\ 4wpf)} = 1.90 \times 10^{-1}$, $p_{(social\ 8wpf)} = 3.05 \times 10^{-1}$, $p_{(isolated\ 2wpf)} = 6.11 \times 10^{-2}$, $p_{(isolated\ 3wpf)} = 1.36 \times 10^{-2}$, $p_{(isolated\ 4wpf)} = 3.41 \times 10^{-2}$, $p_{(isolated\ 8wpf)}$ = NaN (all values equal), Post-hoc Wilcoxon rank-sum test for isolated 3 wpf: $p_{(isolated\ 3wpf,\ wt \Leftrightarrow oxtr^{-/-})} = 4.86 \times 10^{-2}$, $p_{(isolated\ 3wpf,\ wt \Leftrightarrow oxtrl^{-/-})} = 6.59 \times 10^{-2}$, $p_{(isolated\ 3wpf,\ oxtr^{-/-} \Leftrightarrow oxtrl^{-/-})}$ = NaN (all values equal), Post-hoc Wilcoxon rank-sum test for isolated 4 wpf: $p_{(isolated\ 4wpf,\ wt \Leftrightarrow oxtr^{-/-})}$ = NaN (all values equal), $p_{(isolated\ 4wpf,\ wt \Leftrightarrow oxtrl^{-/-})} = 8.25 \times 10^{-2}$, $p_{(isolated\ 4wpf,\ oxtr^{-/-} \Leftrightarrow oxtrl^{-/-})} = 8.25 \times 10^{-2}$,

Number of experimental fish per group: $n_{(social\ wildtype,\ 2\ wpf)} = 15$, $n_{(social\ wildtype,\ 3\ wpf)} = 15$, $n_{(social\ wildtype,\ 4\ wpf)} = 16$, $n_{(social\ wildtype,\ 8\ wpf)} = 13$, $n_{(social\ oxtr^{-/-},\ 2\ wpf)} = 15$, $n_{(social\ oxtr^{-/-},\ 3\ wpf)} = 18$, $n_{(social\ oxtr^{-/-},\ 4\ wpf)} = 12$, $n_{(social\ oxtr^{-/-},\ 8\ wpf)} = 16$, $n_{(social\ oxtrl^{-/-},\ 2\ wpf)} = 18$, $n_{(social\ oxtrl^{-/-},\ 3\ wpf)} = 18$, $n_{(social\ oxtrl^{-/-},\ 4\ wpf)} = 15$, $n_{(social\ oxtrl^{-/-},\ 8\ wpf)} = 11$. $n_{(isolated\ wildtype,\ 2\ wpf)} = 11$, $n_{(isolated\ wildtype,\ 3\ wpf)} = 11$, $n_{(isolated\ wildtype,\ 4\ wpf)} = 9$, $n_{(isolated\ wildtype,\ 8\ wpf)} = 16$, $n_{(isolated\ oxtr^{-/-},\ 2\ wpf)} = 9$, $n_{(isolated\ oxtr^{-/-},\ 3\ wpf)} = 18$, $n_{(isolated\ oxtr^{-/-},\ 4\ wpf)} = 11$, $n_{(isolated\ oxtr^{-/-},\ 8\ wpf)} = 10$, $n_{(isolated\ oxtrl^{-/-},\ 2\ wpf)} = 13$, $n_{(isolated\ oxtrl^{-/-},\ 3\ wpf)} = 12$, $n_{(isolated\ oxtrl^{-/-},\ 4\ wpf)} = 13$, $n_{(isolated\ oxtrl^{-/-},\ 8\ wpf)} = 17$. Each dot represents one experimental fish. Asterisks represent the statistical comparison between genotypes at different ages.

Number of replicates (n) and excluded n can be found in Supplementary Table S1, significance values in Supplementary Table S2. Significance is reported as $*p<0.05$, $**p<0.01$, $***p<0.001$.

color-code: grey = wild-type, green = *oxtr*$^{-/-}$, purple = *oxtrl*$^{-/-}$.

Supplementary Table S1: Number of biological replicates.

For social preference test, n = number of test fish. For shoaling test, n = number of shoals (20 fish per shoal)

**Social preference test** (Figures 1, 2 and S3)												
rearing condition	social											
age group	2 wpf			3 wpf			4 wpf			8 wpf		
genotype group	wildtype	*oxtr*$^{-/-}$	*oxtrl*$^{-/-}$	wildtype	*oxtr*$^{-/-}$	*oxtrl*$^{-/-}$	wildtype	*oxtr*$^{-/-}$	*oxtrl*$^{-/-}$	wildtype	*oxtr*$^{-/-}$	*oxtrl*$^{-/-}$
$n_{(total)}$	18	18	18	18	18	18	18	16	18	18	18	18
excluded$_{(motion)}$	3	3	0	3	0	0	2	4	3	5	2	7
excluded$_{(genotype)}$	0	0	0	0	0	0	0	0	0	0	0	0
$n_{(inAnalysis)}$	15	15	18	15	18	18	16	12	15	13	16	11
rearing condition	isolated											
age group	2 wpf			3 wpf			4 wpf			8 wpf		
genotype group	wildtype	*oxtr*$^{-/-}$	*oxtrl*$^{-/-}$	wildtype	*oxtr*$^{-/-}$	*oxtrl*$^{-/-}$	wildtype	*oxtr*$^{-/-}$	*oxtrl*$^{-/-}$	wildtype	*oxtr*$^{-/-}$	*oxtrl*$^{-/-}$
$n_{(total)}$	16	15	16	17	18	15	15	15	16	18	18	18
excluded$_{(motion)}$	5	6	3	6	0	1	6	2	1	2	8	1
excluded$_{(genotype)}$	0	0	0	0	0	2	0	2	2	0	0	0
$n_{(inAnalysis)}$	11	9	13	11	18	12	9	11	13	16	10	17

**Shoaling test** (Figures 3 and S2)								
age group	4 wpf				8 wpf			
genotype group	*oxtr*$^{+/+}$	*oxtr*$^{-/-}$	*oxtrl*$^{+/+}$	*oxtrl*$^{-/-}$	*oxtr*$^{+/+}$	*oxtr*$^{-/-}$	*oxtrl*$^{+/+}$	*oxtrl*$^{-/-}$
$n_{(total)}$	*26*	*25*	*24*	*29*	*25*	*24*	*25*	*24*
excluded$_{(genotype)}$	*0*	*0*	*9*	*1*	*1*	*3*	*3*	*1*
$n_{(inAnalysis)}$	26	25	15	28	24	21	22	23

qPCR (Figure S1)

	oxtrl level				*oxtr* level			
genotype	*oxtr* $^{+/+}$	*oxtr* $^{-/-}$	*oxtrl* $^{+/+}$	*oxtrl* $^{-/-}$	*oxtr* $^{+/+}$	*oxtr* $^{-/-}$	*oxtrl* $^{+/+}$	*oxtrl* $^{-/-}$
$n_{(inAnalysis)}$	5	6	4	4	4	2	6	4

	4 wpf wildtype						8 wpf wildtype					
	oxt level		*oxtr* level		*oxtrl* level		*oxt* level		*oxtr* level		*oxtrl* level	
rearing	social	isolated	social	isolated	social	isolated	social	isolated	social	isolated	social	isolated
$n_{(inAnalysis)}$	5	6	5	4	5	4	11	10	7	8	7	8

Supplementary Table S2: Significance values of statistical tests (significance is reported as *p<0.05, **p<0.01 and ***p<0.001).

	Kruskal-Wallis test				Wilcoxon rank-sum test				
	groups	effect size (η^2)	p value	signifi-cance	groups	effect size (r)	p value	p value (corrected)	signifi-cance
Fig. 1b	All wildtype, socially reared: 2, 3, 4 and 8 wpf	0.008	9.31×10^{-1}	ns	All wildtype, socially reared: 2 wpf, 3 wpf	0.045	8.03×10^{-1}	8.03×10^{-1}	ns
					All wildtype, socially reared: 2 wpf, 4 wpf	0.096	5.93×10^{-1}	8.03×10^{-1}	ns
					All wildtype, socially reared: 2 wpf, 8 wpf	0.061	7.47×10^{-1}	8.03×10^{-1}	ns
					All wildtype, socially reared: 3 wpf, 4 wpf	0.067	7.07×10^{-1}	8.03×10^{-1}	ns
					All wildtype, socially reared: 3 wpf, 8 wpf	0.074	6.95×10^{-1}	8.03×10^{-1}	ns
					All wildtype, socially reared: 4 wpf, 8 wpf	0.057	7.59×10^{-1}	8.03×10^{-1}	ns
	All *oxtr* $^{-/-}$, socially reared: 2, 3, 4 and 8 wpf	0.168	1.65×10^{-2}	*	All *oxtr* $^{-/-}$, socially reared: 2 wpf, 3 wpf	0.477	6.10×10^{-3}	3.66×10^{-2}	*
					All *oxtr* $^{-/-}$, socially reared: 2 wpf, 4 wpf	0.160	4.07×10^{-1}	6.11×10^{-1}	ns
					All *oxtr* $^{-/-}$, socially reared: 2 wpf, 8 wpf	0.092	6.07×10^{-1}	6.42×10^{-1}	ns

	Kruskal-Wallis test				Wilcoxon rank-sum test				
	groups	η^2	p	sign.	groups	r	p	corr. p	sign.
Fig. 1b	All *oxtr* $^{-/-}$, socially reared: 2, 3, 4 and 8 wpf	0.168	1.65×10^{-2}	*	All *oxtr* $^{-/-}$, socially reared: 3 wpf, 4 wpf	0.381	3.70×10^{-2}	7.40×10^{-2}	ns
					All *oxtr* $^{-/-}$, socially reared: 3 wpf, 8 wpf	0.411	1.65×10^{-2}	4.95×10^{-2}	*
					All *oxtr* $^{-/-}$, socially reared: 4 wpf, 8 wpf	0.088	6.42×10^{-1}	6.42×10^{-1}	ns
	All *oxtrl* $^{-/-}$, socially reared: 2, 3, 4 and 8 wpf	0.365	4.09×10^{-5}	***	All *oxtrl* $^{-/-}$, socially reared: 2 wpf, 3 wpf	0.690	3.49×10^{-5}	2.09×10^{-4}	***
					All *oxtrl* $^{-/-}$, socially reared: 2 wpf, 4 wpf	0.670	1.18×10^{-4}	3.54×10^{-4}	***
					All *oxtrl* $^{-/-}$, socially reared: 2 wpf, 8 wpf	0.541	3.60×10^{-3}	7.20×10^{-3}	**
					All *oxtrl* $^{-/-}$, socially reared: 3 wpf, 4 wpf	0.003	9.86×10^{-1}	9.86×10^{-1}	ns
					All *oxtrl* $^{-/-}$, socially reared: 3 wpf, 8 wpf	0.071	7.01×10^{-1}	9.86×10^{-1}	ns
					All *oxtrl* $^{-/-}$, socially reared: 4 wpf, 8 wpf	0.015	9.38×10^{-1}	9.86×10^{-1}	ns

	Kruskal-Wallis test				Wilcoxon rank-sum test				
	groups	η^2	p	sign.	groups	r	p	corr. p	sign.
Fig. 1c	All 2 wpf, socially reared: wildtype, *oxtr* $^{-/-}$, *oxtrl* $^{-/-}$	0.061	2.25×10^{-1}	ns	All 2 wpf, socially reared: wildtype, *oxtr* $^{-/-}$	0.030	8.68×10^{-1}	8.68×10^{-1}	ns
					All 2 wpf, socially reared: wildtype, *oxtrl* $^{-/-}$	0.252	1.47×10^{-1}	2.36×10^{-1}	ns
					All 2 wpf, socially reared: *oxtr* $^{-/-}$, *oxtrl* $^{-/-}$	0.247	1.57×10^{-1}	2.36×10^{-1}	ns
	All 3 wpf, socially reared: wildtype, *oxtr* $^{-/-}$, *oxtrl* $^{-/-}$	0.147	2.35×10^{-2}	*	All 3 wpf, socially reared: wildtype, *oxtr* $^{-/-}$	0.416	1.68×10^{-2}	2.93×10^{-2}	*
					All 3 wpf, socially reared: wildtype, *oxtrl* $^{-/-}$	0.407	1.95×10^{-2}	2.93×10^{-2}	*
					All 3 wpf, socially reared: *oxtr* $^{-/-}$, *oxtrl* $^{-/-}$	0.064	7.00×10^{-1}	7.00×10^{-1}	ns
	All 4 wpf, socially reared: wildtype, *oxtr* $^{-/-}$, *oxtrl* $^{-/-}$	0.158	3.61×10^{-2}	*	All 4 wpf, socially reared: wildtype, *oxtr* $^{-/-}$	0.018	9.26×10^{-1}	9.26×10^{-1}	ns
					All 4 wpf, socially reared: wildtype, *oxtrl* $^{-/-}$	0.405	2.41×10^{-2}	5.34×10^{-2}	ns
					All 4 wpf, socially reared: *oxtr* $^{-/-}$, *oxtrl* $^{-/-}$	0.404	3.56×10^{-2}	5.34×10^{-2}	ns

	Kruskal-Wallis test				Wilcoxon rank-sum test				
	groups	η^2	p	sign.	groups	r	p	corr. p	sign.
Fig. 1c	All 8 wpf, socially reared: wildtype, *oxtr* $^{-/-}$, *oxtrl* $^{-/-}$	0.064	2.87×10^{-1}	ns	All 8 wpf, socially reared: wildtype, *oxtr* $^{-/-}$	0.024	8.95×10^{-1}	8.95×10^{-1}	ns
					All 8 wpf, socially reared: wildtype, *oxtrl* $^{-/-}$	0.058	2.83×10^{-1}	4.25×10^{-1}	ns
					All 8 wpf, socially reared: *oxtr* $^{-/-}$, *oxtrl* $^{-/-}$	0.304	1.14×10^{-1}	3.42×10^{-1}	ns
Fig 2b	All 2 wpf, isolated reared: wildtype, *oxtr* $^{-/-}$, *oxtrl* $^{-/-}$	0.106	1.85×10^{-1}	ns	All 2 wpf, isolated reared: wildtype, *oxtr* $^{-/-}$	0.357	1.11×10^{-1}	2.22×10^{-1}	ns
					All 2 wpf, isolated reared: wildtype, *oxtrl* $^{-/-}$	0.296	1.48×10^{-1}	2.22×10^{-1}	ns
					All 2 wpf, isolated reared: *oxtr* $^{-/-}$, *oxtrl* $^{-/-}$	0.085	6.89×10^{-1}	6.89×10^{-1}	ns
	All 3 wpf, isolated reared: wildtype, *oxtr* $^{-/-}$, *oxtrl* $^{-/-}$	0.052	3.51×10^{-1}	ns	All 3 wpf, isolated reared: wildtype, *oxtr* $^{-/-}$	0.271	1.44×10^{-1}	4.32×10^{-1}	ns
					All 3 wpf, isolated reared: wildtype, *oxtrl* $^{-/-}$	0.173	4.06×10^{-1}	6.09×10^{-1}	ns
					All 3 wpf, isolated reared: *oxtr* $^{-/-}$, *oxtrl* $^{-/-}$	0.066	7.19×10^{-1}	7.19×10^{-1}	ns

	Kruskal-Wallis test				Wilcoxon rank-sum test				
	groups	η^2	p	sign.	groups	r	p	corr. p	sign.
Fig. 2b	All 4 wpf, isolated reared: wildtype, *oxtr* $^{-/-}$, *oxtrl* $^{-/-}$	0.396	1.80×10^{-3}	**	All 4 wpf, isolated reared: wildtype, *oxtr* $^{-/-}$	0.476	3.34×10^{-2}	5.01×10^{-2}	ns
					All 4 wpf, isolated reared: wildtype, *oxtrl* $^{-/-}$	0.712	8.41×10^{-4}	2.52×10^{-3}	**
					All 4 wpf, isolated reared: *oxtr* $^{-/-}$, *oxtrl* $^{-/-}$	0.343	9.29×10^{-2}	9.29×10^{-2}	ns
	All 8 wpf, isolated reared: wildtype, *oxtr* $^{-/-}$, *oxtrl* $^{-/-}$	0.035	4.82×10^{-1}	ns	All 8 wpf, isolated reared: wildtype, *oxtr* $^{-/-}$	0.088	6.54×10^{-1}	7.07×10^{-1}	ns
					All 8 wpf, isolated reared: wildtype, *oxtrl* $^{-/-}$	0.216	2.14×10^{-1}	6.42×10^{-1}	ns
					All 8 wpf, isolated reared: *oxtr* $^{-/-}$, *oxtrl* $^{-/-}$	0.072	7.07×10^{-1}	7.07×10^{-1}	ns
Fig. 2c	All wildtype, all age groups: social, isolated	0.186	6.70×10^{-3}	**	All 2 wpf wildtype: social, isolated	0.224	2.54×10^{-1}	3.39×10^{-1}	ns
					All 3 wpf wildtype: social, isolated	0.010	9.59×10^{-1}	9.59×10^{-1}	ns
					All 4 wpf wildtype: social, isolated	0.255	2.03×10^{-1}	3.39×10^{-1}	ns
					All 8 wpf wildtype: social, isolated	0.533	4.10×10^{-3}	1.64×10^{-2}	*

	Kruskal-Wallis test				Wilcoxon rank-sum test				
	groups	η^2	p	sign.	groups	r	p	corr. p	sign.
Fig. 2c	All *oxtr* $^{-/-}$, all age groups: social, isolated	0.256	2.00×10^{-4}	***	All 2 wpf *oxtr* $^{-/-}$: social, isolated	0.170	4.09×10^{-1}	4.09×10^{-1}	ns
					All 3 wpf *oxtr* $^{-/-}$: social, isolated	0.158	3.45×10^{-1}	4.09×10^{-1}	ns
					All 4 wpf *oxtr* $^{-/-}$: social, isolated	0.225	2.82×10^{-1}	4.09×10^{-1}	ns
					All 8 wpf *oxtr* $^{-/-}$: social, isolated	0.357	6.90×10^{-2}	2.76×10^{-1}	ns
	All *oxtrl* $^{-/-}$, all age groups: social, isolated	0.435	7.79×10^{-9}	***	All 2 wpf *oxtrl* $^{-/-}$: social, isolated	0.328	6.82×10^{-2}	9.09×10^{-2}	ns
					All 3 wpf *oxtrl* $^{-/-}$: social, isolated	0.171	3.50×10^{-1}	3.50×10^{-1}	ns
					All 4 wpf *oxtrl* $^{-/-}$: social, isolated	0.750	8.94×10^{-5}	3.58×10^{-4}	***
					All 8 wpf *oxtrl* $^{-/-}$: social, isolated	0.507	7.30×10^{-3}	1.46×10^{-2}	*
Fig. 3a	Nearest-neighbor distance (NND), all 4 wpf, socially reared: *oxtr* $^{+/+}$, *oxtr* $^{-/-}$, *oxtrl* $^{+/+}$, *oxtrl* $^{-/-}$	0.080	1.69×10^{-2}	*	All 4 wpf, socially reared: *oxtr* $^{+/+}$, *oxtr* $^{-/-}$	0.011	4.74×10^{-1}	4.74×10^{-1}	ns
					All 4 wpf, socially reared: *oxtrl* $^{+/+}$, *oxtrl* $^{-/-}$	0.124	2.11×10^{-1}	3.17×10^{-1}	ns
					All 4 wpf, socially reared: *oxtr* $^{+/+}$, *oxtrl* $^{+/+}$	0.199	1.04×10^{-1}	3.12×10^{-1}	ns
	See page below	0.229	4.72×10^{-5}	***	All 8 wpf, socially reared: *oxtr* $^{+/+}$, *oxtr* $^{-/-}$	0.488	5.48×10^{-4}	1.64×10^{-3}	**

	Kruskal-Wallis test				Wilcoxon rank-sum test				
	groups	η^2	p	sign.	groups	r	p	corr. p	sign.
Fig. 3a	NND, all 8 wpf, socially reared: *oxtr* $^{+/+}$, *oxtr* $^{-/-}$, *oxtrl* $^{+/+}$, *oxtrl* $^{-/-}$	0.229	4.72×10^{-5}	***	All 8 wpf, socially reared: *oxtrl* $^{+/+}$, *oxtrl* $^{-/-}$	0.288	2.75×10^{-2}	4.13×10^{-2}	*
					All 8 wpf, socially reared: *oxtr* $^{+/+}$, *oxtrl* $^{+/+}$	0.240	5.30×10^{-2}	5.30×10^{-2}	ns
Fig. 3b	Inter-individual distance, all 4 wpf, socially reared: *oxtr* $^{+/+}$, *oxtr* $^{-/-}$, *oxtrl* $^{+/+}$, *oxtrl* $^{-/-}$	0.017	2.07×10^{-1}	ns	All 4 wpf, socially reared: *oxtr* $^{+/+}$, *oxtr* $^{-/-}$	0.042	3.85×10^{-1}	4.09×10^{-1}	ns
					All 4 wpf, socially reared: *oxtrl* $^{+/+}$, *oxtrl* $^{-/-}$	0.210	8.64×10^{-2}	2.59×10^{-1}	ns
					All 4 wpf, socially reared: *oxtr* $^{+/+}$, *oxtrl* $^{+/+}$	0.038	4.09×10^{-1}	4.09×10^{-1}	ns
	Inter-individual distance, all 8 wpf, socially reared: *oxtr* $^{+/+}$, *oxtr* $^{-/-}$, *oxtrl* $^{+/+}$, *oxtrl* $^{-/-}$	0.133	2.40×10^{-3}	**	All 8 wpf, socially reared: *oxtr* $^{+/+}$, *oxtr* $^{-/-}$	0.390	4.59×10^{-3}	1.38×10^{-2}	*
					All 8 wpf, socially reared: *oxtrl* $^{+/+}$, *oxtrl* $^{-/-}$	0.332	1.34×10^{-2}	2.01×10^{-2}	*
					All 8 wpf, socially reared: *oxtr* $^{+/+}$, *oxtrl* $^{+/+}$	0.204	8.47×10^{-2}	8.47×10^{-2}	ns
Fig. 3c	See page below	0.006	4.79×10^{-1}	ns	All 4 wpf, socially reared: *oxtr* $^{+/+}$, *oxtr* $^{-/-}$	0.011	4.74×10^{-1}	4.74×10^{-1}	ns

	Kruskal-Wallis test				Wilcoxon rank-sum test				
	groups	η^2	p	sign.	groups	r	p	corr. p	sign.
Fig. 3c	Farthest-neighbor distance, all 4 wpf, socially reared: $oxtr^{+/+}$, $oxtr^{-/-}$, $oxtrl^{+/+}$, $oxtrl^{-/-}$	0.006	4.79×10^{-1}	ns	All 4 wpf, socially reared: $oxtrl^{+/+}$, $oxtrl^{-/-}$	0.218	7.86×10^{-2}	1.29×10^{-1}	ns
					All 4 wpf, socially reared: $oxtr^{+/+}$, $oxtrl^{+/+}$	0.216	8.58×10^{-2}	1.29×10^{-1}	ns
	Farthest-neighbor distance, all 8 wpf, socially reared: $oxtr^{+/+}$, $oxtr^{-/-}$, $oxtrl^{+/+}$, $oxtrl^{-/-}$	0.130	2.65×10^{-3}	**	All 8 wpf, socially reared: $oxtr^{+/+}$, $oxtr^{-/-}$	0.359	8.19×10^{-3}	1.91×10^{-2}	*
					All 8 wpf, socially reared: $oxtrl^{+/+}$, $oxtrl^{-/-}$	0.335	1.27×10^{-2}	1.91×10^{-2}	*
					All 8 wpf, socially reared: $oxtr^{+/+}$, $oxtrl^{+/+}$	0.240	5.30×10^{-2}	5.30×10^{-2}	ns
Fig. 3d	Variance explained, All 4wpf, socially reared: $oxtr^{+/+}$, $oxtr^{-/-}$, $oxtrl^{+/+}$, $oxtrl^{-/-}$	0.020	5.64×10^{-1}	ns	All 4 wpf, socially reared: $oxtr^{+/+}$, $oxtr^{-/-}$	0.020	8.88×10^{-1}	8.88×10^{-1}	ns
					All 4 wpf, socially reared: $oxtrl^{+/+}$, $oxtrl^{-/-}$	0.214	1.60×10^{-1}	4.80×10^{-1}	ns
					All 4 wpf, socially reared: $oxtr^{+/+}$, $oxtrl^{+/+}$	0.120	4.43×10^{-1}	6.65×10^{-1}	ns
	Variance explained, All 8wpf, socially reared: $oxtr^{+/+}$, $oxtr^{-/-}$, $oxtrl^{+/+}$, $oxtrl^{-/-}$	0.194	7.00×10^{-4}	**	All 8 wpf, socially reared: $oxtr^{+/+}$, $oxtr^{-/-}$	0.357	1.65×10^{-2}	4.95×10^{-2}	*
					All 8 wpf, socially reared: $oxtrl^{+/+}$, $oxtrl^{-/-}$	0.293	4.95×10^{-2}	4.95×10^{-2}	*

	Kruskal-Wallis test				Wilcoxon rank-sum test				
	groups	η^2	p	sign.	groups	r	p	corr. p	sign.
Fig. 3d	See previous page	0.194	7.00×10^{-4}	**	All 8 wpf, socially reared: *oxtr* $^{+/+}$, *oxtrl* $^{+/+}$	0.314	3.31×10^{-2}	4.95×10^{-2}	*
Fig. S1b	All 8 wpf, socially reared: *oxtr* $^{+/+}$, *oxtr* $^{-/-}$, *oxtrl* $^{+/+}$, *oxtrl* $^{-/-}$	0.034	8.80×10^{-1}	ns	All 8 wpf, socially reared: *oxtr* $^{+/+}$, *oxtr* $^{-/-}$	0.140	9.31×10^{-1}	9.31×10^{-1}	ns
					All 8 wpf, socially reared: *oxtrl* $^{+/+}$, *oxtrl* $^{-/-}$	0.179	6.10×10^{-1}	9.31×10^{-1}	ns
Fig. S1c	*oxt* level, all wildtype, all age groups: social, isolated	0.542	8.00×10^{-4}	***	All 4 wpf wildtype: social, isolated	0.365	3.29×10^{-1}	3.29×10^{-1}	ns
					All 8 wpf wildtype: social, isolated	0.511	6.70×10^{-3}	1.34×10^{-2}	*
					All wildtype, socially reared: 4 wpf, 8 wpf	0.643	4.58×10^{-4}	1.83×10^{-3}	**
					All wildtype, isolated reared: 4 wpf, 8 wpf	0.323	3.13×10^{-1}	3.29×10^{-1}	ns
Fig. S1d	*oxtr* level, all wildtype, all age groups: social, isolated	0.005	9.90×10^{-1}	ns	All 4 wpf wildtype: social, isolated	0.071	9.05×10^{-1}	9.33×10^{-1}	ns
					All 8 wpf wildtype: social, isolated	0.084	7.79×10^{-1}	9.33×10^{-1}	ns

	Kruskal-Wallis test				Wilcoxon rank-sum test				
	groups	η^2	p	sign.	groups	r	p	corr. p	sign.
Fig. S1d	*oxtl* level, all wildtype, all age groups: social, isolated	0.005	9.90×10^{-1}	ns	All wildtype, socially reared: 4 wpf, 8 wpf	0.103	8.76×10^{-1}	9.33×10^{-1}	ns
					All wildtype, isolated reared: 4 wpf, 8 wpf	0.047	9.33×10^{-1}	9.33×10^{-1}	ns
Fig. S1e	*oxtrl* level, all wildtype, all age groups: Social, isolated	0.004	9.91×10^{-1}	ns	All 4 wpf wildtype: social, isolated	0.088	9.05×10^{-1}	9.05×10^{-1}	ns
					All 8 wpf wildtype: social, isolated	0.083	7.79×10^{-1}	9.05×10^{-1}	ns
					All wildtype, socially reared: 4 wpf, 8 wpf	0.001	8.76×10^{-1}	9.05×10^{-1}	ns
					All wildtype, isolated reared: 4 wpf, 8 wpf	0.166	8.08×10^{-1}	9.05×10^{-1}	ns
Fig. S2b	Cumulative shoal distance, all 4 wpf, socially reared, *oxtr* $^{+/+}$, *oxtr* $^{-/-}$, *oxtrl* $^{+/+}$, *oxtrl* $^{-/-}$	0.200	2.00×10^{-4}	**	All 4 wpf, socially reared: *oxtr* $^{+/+}$, *oxtr* $^{-/-}$	0.270	5.34×10^{-2}	8.01×10^{-2}	ns
					All 4 wpf, socially reared: *oxtrl* $^{+/+}$, *oxtrl* $^{-/-}$	0.115	4.52×10^{-1}	4.52×10^{-1}	ns
					All 4 wpf, socially reared: *oxtr* $^{+/+}$, *oxtrl* $^{+/+}$	0.370	1.79×10^{-2}	5.37×10^{-2}	ns

	Kruskal-Wallis test				Wilcoxon rank-sum test				
	groups	η^2	p	sign.	groups	r	p	corr. p	sign.
Fig. S2b	Cumulative shoal distance, all 8 wpf, socially reared, *oxtr* $^{+/+}$, *oxtr* $^{-/-}$, *oxtrl* $^{+/+}$, *oxtrl* $^{-/-}$	0.087	5.25×10^{-2}	ns	All 8 wpf, socially reared: *oxtr* $^{+/+}$, *oxtr* $^{-/-}$	0.280	6.05×10^{-2}	1.82×10^{-1}	ns
					All 8 wpf, socially reared: *oxtrl* $^{+/+}$, *oxtrl* $^{-/-}$	0.012	9.37×10^{-1}	9.37×10^{-1}	ns
					All 8 wpf, socially reared: *oxtr* $^{+/+}$, *oxtrl* $^{+/+}$	0.212	1.50×10^{-1}	2.25×10^{-1}	ns
Fig. S2c	Boldness, all 4 wpf, socially reared, *oxtr* $^{+/+}$, *oxtr* $^{-/-}$, *oxtrl* $^{+/+}$, *oxtrl* $^{-/-}$	0.170	1.20×10^{-3}	**	All 4 wpf, socially reared: *oxtr* $^{+/+}$, *oxtr* $^{-/-}$	0.133	3.41×10^{-1}	3.66×10^{-1}	ns
					All 4 wpf, socially reared: *oxtrl* $^{+/+}$, *oxtrl* $^{-/-}$	0.138	3.66×10^{-1}	3.66×10^{-1}	ns
					All 4 wpf, socially reared: *oxtr* $^{+/+}$, *oxtrl* $^{+/+}$	0.319	4.10×10^{-2}	1.23×10^{-1}	ns
	Boldness, all 8 wpf, socially reared, *oxtr* $^{+/+}$, *oxtr* $^{-/-}$, *oxtrl* $^{+/+}$, *oxtrl* $^{-/-}$	0.295	8.54×10^{-6}	***	All 8 wpf, socially reared: *oxtr* $^{+/+}$, *oxtr* $^{-/-}$	0.185	2.15×10^{-1}	2.71×10^{-1}	ns
					All 8 wpf, socially reared: *oxtrl* $^{+/+}$, *oxtrl* $^{-/-}$	0.164	2.71×10^{-1}	2.71×10^{-1}	ns
					All 8 wpf, socially reared: *oxtr* $^{+/+}$, *oxtrl* $^{+/+}$	0.413	5.10×10^{-2}	1.53×10^{-1}	ns

	Kruskal-Wallis test				Wilcoxon rank-sum test				
	groups	η^2	p	sign.	groups	r	p	corr. p	sign.
Fig. S3a	All 2 wpf, socially reared: wildtype, *oxtr* $^{-/-}$, *oxtrl* $^{-/-}$	0.309	3.49×10^{-4}	***	All 2 wpf, socially reared: wildtype, *oxtr* $^{-/-}$	0.674	2.23×10^{-4}	6.69×10^{-4}	***
					All 2 wpf, socially reared: wildtype, *oxtrl* $^{-/-}$	0.526	2.54×10^{-3}	3.81×10^{-3}	**
					All 2 wpf, socially reared: *oxtr* $^{-/-}$, *oxtrl* $^{-/-}$	0.186	2.86×10^{-1}	2.86×10^{-1}	ns
	All 3 wpf, socially reared: wildtype, *oxtr* $^{-/-}$, *oxtrl* $^{-/-}$	0.314	1.97×10^{-4}	***	All 3 wpf, socially reared: wildtype, *oxtr* $^{-/-}$	0.645	2.11×10^{-4}	6.33×10^{-4}	***
					All 3 wpf, socially reared: wildtype, *oxtrl* $^{-/-}$	0.601	5.55×10^{-4}	8.33×10^{-4}	***
					All 3 wpf, socially reared: *oxtr* $^{-/-}$, *oxtrl* $^{-/-}$	0.066	6.92×10^{-1}	6.92×10^{-1}	ns
	All 4 wpf, socially reared: wildtype, *oxtr* $^{-/-}$, *oxtrl* $^{-/-}$	0.218	4.71×10^{-3}	**	All 4 wpf, socially reared: wildtype, *oxtr* $^{-/-}$	0.224	2.36×10^{-1}	2.36×10^{-1}	ns
					All 4 wpf, socially reared: wildtype, *oxtrl* $^{-/-}$	0.515	4.16×10^{-3}	1.25×10^{-2}	*
					All 4 wpf, socially reared: *oxtr* $^{-/-}$, *oxtrl* $^{-/-}$	0.474	1.37×10^{-2}	2.06×10^{-2}	*

	Kruskal-Wallis test				Wilcoxon rank-sum test				
	groups	η^2	p	sign.	groups	r	p	corr. p	sign.
Fig. S3a	All 8 wpf, socially reared: wildtype, *oxtr* $^{-/-}$, *oxtrl* $^{-/-}$	0.069	9.84×10^{-2}	ns	All 8 wpf, socially reared: wildtype, *oxtr* $^{-/-}$	0.354	5.64×10^{-2}	1.64×10^{-1}	ns
					All 8 wpf, socially reared: wildtype, *oxtrl* $^{-/-}$	0.083	6.85×10^{-1}	6.85×10^{-1}	ns
					All 8 wpf, socially reared: *oxtr* $^{-/-}$, *oxtrl* $^{-/-}$	0.309	1.09×10^{-1}	1.64×10^{-1}	ns
	All 2 wpf, isolation reared: wildtype, *oxtr* $^{-/-}$, *oxtrl* $^{-/-}$	0.464	3.48×10^{-4}	***	All 2 wpf, isolation reared: wildtype, *oxtr* $^{-/-}$	0.747	8.29×10^{-4}	2.49×10^{-3}	**
					All 2 wpf, isolation reared: wildtype, *oxtrl* $^{-/-}$	0.627	2.14×10^{-3}	3.21×10^{-3}	**
					All 2 wpf, isolation reared: *oxtr* $^{-/-}$, *oxtrl* $^{-/-}$	0.379	8.25×10^{-2}	8.25×10^{-2}	ns
	All 3 wpf, isolation reared: wildtype, *oxtr* $^{-/-}$, *oxtrl* $^{-/-}$	0.492	3.22×10^{-5}	***	All 3 wpf, isolation reared: wildtype, *oxtr* $^{-/-}$	0.789	2.16×10^{-5}	6.48×10^{-5}	***
					All 3 wpf, isolation reared: wildtype, *oxtrl* $^{-/-}$	0.379	6.94×10^{-2}	6.94×10^{-2}	ns
					All 3 wpf, isolation reared: *oxtr* $^{-/-}$, *oxtrl* $^{-/-}$	0.506	5.56×10^{-3}	8.34×10^{-3}	**

	Kruskal-Wallis test				Wilcoxon rank-sum test				
	groups	η^2	p	sign.	groups	r	p	corr. p	sign.
Fig. S3a	All 4 wpf, isolation reared: wildtype, *oxtr* $^{-/-}$, *oxtrl* $^{-/-}$	0.003	3.86×10^{-1}	ns	All 4 wpf, isolation reared: wildtype, *oxtr* $^{-/-}$	0.085	7.04×10^{-1}	7.04×10^{-1}	ns
					All 4 wpf, isolation reared: wildtype, *oxtrl* $^{-/-}$	0.185	3.85×10^{-1}	5.78×10^{-1}	ns
					All 4 wpf, isolation reared: *oxtr* $^{-/-}$, *oxtrl* $^{-/-}$	0.260	2.02×10^{-1}	5.78×10^{-1}	ns
	All 8 wpf, isolation reared: wildtype, *oxtr* $^{-/-}$, *oxtrl* $^{-/-}$	0.015	2.70×10^{-1}	ns	All 8 wpf, isolation reared: wildtype, *oxtr* $^{-/-}$	0.181	3.56×10^{-1}	5.28×10^{-1}	ns
					All 8 wpf, isolation reared: wildtype, *oxtrl* $^{-/-}$	0.110	5.28×10^{-1}	5.28×10^{-1}	ns
					All 8 wpf, isolation reared: *oxtr* $^{-/-}$, *oxtrl* $^{-/-}$	0.314	1.03×10^{-1}	3.09×10^{-1}	ns
Fig. S3b	All 2 wpf, socially reared: wildtype, *oxtr* $^{-/-}$, *oxtrl* $^{-/-}$	0.230	2.08×10^{-3}	**	All 2 wpf, socially reared: wildtype, *oxtr* $^{-/-}$	0.682	1.89×10^{-4}	5.67×10^{-4}	***
					All 2 wpf, socially reared: wildtype, *oxtrl* $^{-/-}$	0.356	4.11×10^{-2}	6.17×10^{-2}	ns
					All 2 wpf, socially reared: *oxtr* $^{-/-}$, *oxtrl* $^{-/-}$	0.167	3.38×10^{-1}	3.38×10^{-1}	ns

	Kruskal-Wallis test				Wilcoxon rank-sum test				
	groups	η^2	p	sign.	groups	r	p	corr. p	sign.
Fig. S3b	All 3 wpf, socially reared: wildtype, *oxtr* $^{-/-}$, *oxtrl* $^{-/-}$	0.256	7.97×10^{-4}	***	All 3 wpf, socially reared: wildtype, *oxtr* $^{-/-}$	0.450	9.70×10^{-3}	1.46×10^{-2}	*
					All 3 wpf, socially reared: wildtype, *oxtrl* $^{-/-}$	0.645	2.11×10^{-4}	6.33×10^{-4}	***
					All 3 wpf, socially reared: *oxtr* $^{-/-}$, *oxtrl* $^{-/-}$	0.171	3.04×10^{-1}	3.04×10^{-1}	ns
	All 4 wpf, socially reared: wildtype, *oxtr* $^{-/-}$, *oxtrl* $^{-/-}$	0.010	3.04×10^{-1}	ns	All 4 wpf, socially reared: wildtype, *oxtr* $^{-/-}$	0.224	2.37×10^{-1}	3.56×10^{-1}	ns
					All 4 wpf, socially reared: wildtype, *oxtrl* $^{-/-}$	0.018	9.21×10^{-1}	9.21×10^{-1}	ns
					All 4 wpf, socially reared: *oxtr* $^{-/-}$, *oxtrl* $^{-/-}$	0.286	1.37×10^{-1}	3.56×10^{-1}	ns
	All 8 wpf, socially reared: wildtype, *oxtr* $^{-/-}$, *oxtrl* $^{-/-}$	0.032	6.61×10^{-1}	ns	All 8 wpf, socially reared: wildtype, *oxtr* $^{-/-}$	0.110	5.54×10^{-1}	7.86×10^{-1}	ns
					All 8 wpf, socially reared: wildtype, *oxtrl* $^{-/-}$	0.177	3.85×10^{-1}	7.86×10^{-1}	ns
					All 8 wpf, socially reared: *oxtr* $^{-/-}$, *oxtrl* $^{-/-}$	0.052	7.86×10^{-1}	7.86×10^{-1}	ns

	Kruskal-Wallis test				Wilcoxon rank-sum test				
	groups	η^2	p	sign.	groups	r	p	corr. p	sign.
Fig. S3b	All 2 wpf, isolation reared: wildtype, *oxtr*$^{-/-}$, *oxtrl*$^{-/-}$	0.581	6.03×10^{-5}	***	All 2 wpf, isolation reared: wildtype, *oxtr*$^{-/-}$	0.815	2.66×10^{-4}	7.98×10^{-4}	***
					All 2 wpf, isolation reared: wildtype, *oxtrl*$^{-/-}$	0.509	1.27×10^{-2}	1.27×10^{-2}	*
					All 2 wpf, isolation reared: *oxtr*$^{-/-}$, *oxtrl*$^{-/-}$	0.699	1.30×10^{-3}	1.95×10^{-3}	**
	All 3 wpf, isolation reared: wildtype, *oxtr*$^{-/-}$, *oxtrl*$^{-/-}$	0.564	8.11×10^{-6}	***	All 3 wpf, isolation reared: wildtype, *oxtr*$^{-/-}$	0.755	4.75×10^{-5}	1.43×10^{-4}	***
					All 3 wpf, isolation reared: wildtype, *oxtrl*$^{-/-}$	0.558	7.40×10^{-3}	7.40×10^{-3}	**
					All 3 wpf, isolation reared: *oxtr*$^{-/-}$, *oxtrl*$^{-/-}$	0.607	8.90×10^{-4}	1.34×10^{-3}	**
	All 4 wpf, isolation reared: wildtype, *oxtr*$^{-/-}$, *oxtrl*$^{-/-}$	0.075	1.19×10^{-1}	ns	All 4 wpf, isolation reared: wildtype, *oxtr*$^{-/-}$	0.136	5.43×10^{-1}	5.43×10^{-1}	ns
					All 4 wpf, isolation reared: wildtype, *oxtrl*$^{-/-}$	0.384	7.14×10^{-2}	1.98×10^{-1}	ns
					All 4 wpf, isolation reared: *oxtr*$^{-/-}$, *oxtrl*$^{-/-}$	0.307	1.32×10^{-1}	1.98×10^{-1}	ns

	Kruskal-Wallis test				Wilcoxon rank-sum test				
	groups	η^2	p	sign.	groups	r	p	corr. p	sign.
Fig. S3b	All 8 wpf, isolation reared: wildtype, *oxtr* $^{-/-}$, *oxtrl* $^{-/-}$	0.170	1.23×10^{-2}	*	All 8 wpf, isolation reared: wildtype, *oxtr* $^{-/-}$	0.563	4.10×10^{-3}	1.23×10^{-2}	*
					All 8 wpf, isolation reared: wildtype, *oxtrl* $^{-/-}$	0.097	5.77×10^{-1}	5.77×10^{-1}	ns
					All 8 wpf, isolation reared: *oxtr* $^{-/-}$, *oxtrl* $^{-/-}$	0.440	2.23×10^{-2}	3.35×10^{-2}	*
Fig. S3c	All 2 wpf, socially reared: wildtype, *oxtr* $^{-/-}$, *oxtrl* $^{-/-}$	0.077	6.57×10^{-2}	ns	All 2 wpf, socially reared: wildtype, *oxtr* $^{-/-}$	0.097	5.94×10^{-1}	5.94×10^{-1}	ns
					All 2 wpf, socially reared: wildtype, *oxtrl* $^{-/-}$	0.288	9.82×10^{-2}	2.95×10^{-1}	ns
					All 2 wpf, socially reared: *oxtr* $^{-/-}$, *oxtrl* $^{-/-}$	0.360	3.85×10^{-1}	5.78×10^{-1}	ns
	All 3 wpf, socially reared: wildtype, *oxtr* $^{-/-}$, *oxtrl* $^{-/-}$	0.014	2.71×10^{-1}	ns	All 3 wpf, socially reared: wildtype, *oxtr* $^{-/-}$	0.067	7.01×10^{-1}	7.01×10^{-1}	ns
					All 3 wpf, socially reared: wildtype, *oxtrl* $^{-/-}$	0.244	1.61×10^{-1}	3.48×10^{-1}	ns
					All 3 wpf, socially reared: *oxtr* $^{-/-}$, *oxtrl* $^{-/-}$	0.199	2.32×10^{-1}	3.48×10^{-1}	ns

	Kruskal-Wallis test				Wilcoxon rank-sum test				
	groups	η^2	p	sign.	groups	r	p	corr. p	sign.
Fig. S3c	All 4 wpf, socially reared: wildtype, *oxtr* $^{-/-}$, *oxtrl* $^{-/-}$	0.089	6.25×10^{-2}	ns	All 4 wpf, socially reared: wildtype, *oxtr* $^{-/-}$	0.205	2.79×10^{-1}	2.79×10^{-1}	ns
					All 4 wpf, socially reared: wildtype, *oxtrl* $^{-/-}$	0.384	3.26×10^{-2}	9.77×10^{-2}	ns
					All 4 wpf, socially reared: *oxtr* $^{-/-}$, *oxtrl* $^{-/-}$	0.236	2.21×10^{-1}	2.79×10^{-1}	ns
	All 8 wpf, socially reared: wildtype, *oxtr* $^{-/-}$, *oxtrl* $^{-/-}$	0.012	2.91×10^{-1}	ns	All 8 wpf, socially reared: wildtype, *oxtr* $^{-/-}$	0.241	1.95×10^{-1}	2.93×10^{-1}	ns
					All 8 wpf, socially reared: wildtype, *oxtrl* $^{-/-}$	0.321	1.16×10^{-1}	2.93×10^{-1}	ns
					All 8 wpf, socially reared: *oxtr* $^{-/-}$, *oxtrl* $^{-/-}$	0.069	7.19×10^{-1}	7.19×10^{-1}	ns
	All 2 wpf, isolation reared: wildtype, *oxtr* $^{-/-}$, *oxtrl* $^{-/-}$	0.006	4.05×10^{-1}	ns	All 2 wpf, isolation reared: wildtype, *oxtr* $^{-/-}$	0.303	1.76×10^{-1}	5.28×10^{-1}	ns
					All 2 wpf, isolation reared: wildtype, *oxtrl* $^{-/-}$	0.137	5.03×10^{-1}	5.84×10^{-1}	ns
					All 2 wpf, isolation reared: *oxtr* $^{-/-}$, *oxtrl* $^{-/-}$	0.119	5.84×10^{-1}	5.84×10^{-1}	ns

	Kruskal-Wallis test				Wilcoxon rank-sum test				
	groups	η^2	p	sign.	groups	r	p	corr. p	sign.
Fig. S3c	All 3 wpf, isolation reared: wildtype, *oxtr* $^{-/-}$, *oxtrl* $^{-/-}$	0.010	4.48×10^{-1}	ns	All 3 wpf, isolation reared: wildtype, *oxtr* $^{-/-}$	0.224	2.27×10^{-1}	3.71×10^{-1}	ns
					All 3 wpf, isolation reared: wildtype, *oxtrl* $^{-/-}$	0	1	1	ns
					All 3 wpf, isolation reared: *oxtr* $^{-/-}$, *oxtrl* $^{-/-}$	0.211	2.47×10^{-1}	3.71×10^{-1}	ns
	All 4 wpf, isolation reared: wildtype, *oxtr* $^{-/-}$, *oxtrl* $^{-/-}$	0.030	5.76×10^{-1}	ns	All 4 wpf, isolation reared: wildtype, *oxtr* $^{-/-}$	0.225	3.15×10^{-1}	6.05×10^{-1}	ns
					All 4 wpf, isolation reared: wildtype, *oxtrl* $^{-/-}$	0.029	8.94×10^{-1}	8.94×10^{-1}	ns
					All 4 wpf, isolation reared: *oxtr* $^{-/-}$, *oxtrl* $^{-/-}$	0.171	4.03×10^{-1}	6.05×10^{-1}	ns
	All 8 wpf, isolation reared: wildtype, *oxtr* $^{-/-}$, *oxtrl* $^{-/-}$	NaN	NaN	NaN	All 8 wpf, isolation reared: wildtype, *oxtr* $^{-/-}$	NaN	NaN	NaN	NaN
					All 8 wpf, isolation reared: wildtype, *oxtrl* $^{-/-}$	NaN	NaN	NaN	NaN
					All 8 wpf, isolation reared: *oxtr* $^{-/-}$, *oxtrl* $^{-/-}$	NaN	NaN	NaN	NaN

	Kruskal-Wallis test				Wilcoxon rank-sum test				
	groups	η^2	p	sign.	groups	r	p	corr. p	sign.
Fig. S3d	All 2 wpf, socially reared: wildtype, *oxtr* $^{-/-}$, *oxtrl* $^{-/-}$	0.086	5.36×10^{-2}	ns	All 2 wpf, socially reared: wildtype, *oxtr* $^{-/-}$	0	1	1	ns
					All 2 wpf, socially reared: wildtype, *oxtrl* $^{-/-}$	0.349	4.51×10^{-2}	7.41×10^{-2}	ns
					All 2 wpf, socially reared: *oxtr* $^{-/-}$, *oxtrl* $^{-/-}$	0.342	4.94×10^{-2}	7.41×10^{-2}	ns
	All 3 wpf, socially reared: wildtype, *oxtr* $^{-/-}$, *oxtrl* $^{-/-}$	0.011	4.79×10^{-1}	ns	All 3 wpf, socially reared: wildtype, *oxtr* $^{-/-}$	0.106	5.43×10^{-1}	6.48×10^{-1}	ns
					All 3 wpf, socially reared: wildtype, *oxtrl* $^{-/-}$	0.080	6.48×10^{-1}	6.48×10^{-1}	ns
					All 3 wpf, socially reared: *oxtr* $^{-/-}$, *oxtrl* $^{-/-}$	0.202	2.25×10^{-1}	6.48×10^{-1}	ns
	All 4 wpf, socially reared: wildtype, *oxtr* $^{-/-}$, *oxtrl* $^{-/-}$	0.033	1.90×10^{-1}	ns	All 4 wpf, socially reared: wildtype, *oxtr* $^{-/-}$	0.274	1.47×10^{-1}	2.51×10^{-1}	ns
					All 4 wpf, socially reared: wildtype, *oxtrl* $^{-/-}$	0	1	1	ns
					All 4 wpf, socially reared: *oxtr* $^{-/-}$, *oxtrl* $^{-/-}$	0.266	1.67×10^{-1}	2.51×10^{-1}	ns

	Kruskal-Wallis test				Wilcoxon rank-sum test				
	groups	η^2	p	sign.	groups	r	p	corr. p	sign.
Fig. S3d	All 8 wpf, socially reared: wildtype, *oxtr* $^{-/-}$, *oxtrl* $^{-/-}$	0.010	3.05×10^{-1}	ns	All 8 wpf, socially reared: wildtype, *oxtr* $^{-/-}$	0.232	2.12×10^{-1}	3.18×10^{-1}	ns
					All 8 wpf, socially reared: wildtype, *oxtrl* $^{-/-}$	0.308	1.31×10^{-1}	3.18×10^{-1}	ns
					All 8 wpf, socially reared: *oxtr* $^{-/-}$, *oxtrl* $^{-/-}$	0.084	6.61×10^{-1}	6.61×10^{-1}	ns
	All 2 wpf, isolation reared: wildtype, *oxtr* $^{-/-}$, *oxtrl* $^{-/-}$	0.120	6.11×10^{-2}	ns	All 2 wpf, isolation reared: wildtype, *oxtr* $^{-/-}$	0.303	1.76×10^{-1}	2.22×10^{-1}	ns
					All 2 wpf, isolation reared: wildtype, *oxtrl* $^{-/-}$	0.249	2.22×10^{-1}	2.22×10^{-1}	ns
					All 2 wpf, isolation reared: *oxtr* $^{-/-}$, *oxtrl* $^{-/-}$	0.478	2.86×10^{-2}	8.58×10^{-2}	ns
	All 3 wpf, isolation reared: wildtype, *oxtr* $^{-/-}$, *oxtrl* $^{-/-}$	0.174	1.36×10^{-2}	*	All 3 wpf, isolation reared: wildtype, *oxtr* $^{-/-}$	0.418	2.43×10^{-2}	4.86×10^{-2}	*
					All 3 wpf, isolation reared: wildtype, *oxtrl* $^{-/-}$	0.384	6.59×10^{-2}	6.59×10^{-2}	ns
					All 3 wpf, isolation reared: *oxtr* $^{-/-}$, *oxtrl* $^{-/-}$	NaN	NaN	NaN	NaN

	Kruskal-Wallis test				Wilcoxon rank-sum test				
	groups	η^2	p	sign.	groups	r	p	corr. p	sign.
Fig. S3d	All 4 wpf, isolation reared: wildtype, *oxtr* $^{-/-}$, *oxtrl* $^{-/-}$	0.159	3.41×10^{-2}	*	All 4 wpf, isolation reared: wildtype, *oxtr* $^{-/-}$	NaN	NaN	NaN	NaN
					All 4 wpf, isolation reared: wildtype, *oxtrl* $^{-/-}$	0.370	8.25×10^{-2}	8.25×10^{-2}	ns
					All 4 wpf, isolation reared: *oxtr* $^{-/-}$, *oxtrl* $^{-/-}$	0.392	5.11×10^{-2}	8.25×10^{-2}	ns
	All 8 wpf, isolation reared: wildtype, *oxtr* $^{-/-}$, *oxtrl* $^{-/-}$	NaN	NaN	NaN	All 8 wpf, isolation reared: wildtype, *oxtr* $^{-/-}$	NaN	NaN	NaN	NaN
					All 8 wpf, isolation reared: wildtype, *oxtrl* $^{-/-}$	NaN	NaN	NaN	NaN
					All 8 wpf, isolation reared: *oxtr* $^{-/-}$, *oxtrl* $^{-/-}$	NaN	NaN	NaN	NaN
